# Prominent Cold Nodule in Multinodular Goiter Revealed to Be Thyrolipoma: A Case Report

**Published:** 2016-12-24

**Authors:** Maryam Kadivar, Parnian Kheirkhah Rahimabad, Sareh Salarinejad

**Affiliations:** 1 *Dept. of Pathology, Rasoul Akram Hospital, Iran University of Medical Sciences, Tehran, Iran*; 2 *School of Medicine, Iran University of Medical Sciences, Tehran, Iran*

**Keywords:** Thyrolipoma, Thyroid Gland, Adenolipoma, Histopathology

## Abstract

The presence of adipose tissue in the thyroid gland is a rare finding. Thyrolipoma or adenolipoma of the thyroid is a benign, encapsulated lesion of the thyroid composed of variable amounts of fat and glandular elements. This report presents a case of thyrolipoma in a 69-yr-old female presenting with neck swelling and respiratory distress. Differential diagnosis of the fat-containing thyroid lesion is also presented. Differentiation of the condition from similar lesions is necessary for accurate diagnosis of thyrolipoma**.**

## Introduction

Thyrolipomas are rare, benign, encapsulated lesions of the thyroid gland containing mature adipose tissue (1-3). Patients with thyrolipoma are usually euthyroid and present with neck enlargement with or without symptoms of compression (1, 4). Differentiating thyrolipoma from its mimics is important in two ways. First, the rarity and diversity of thyroid fat-containing lesions make the differential diagnosis challenging (4). Second, differential diagnosis also includes malignant lesions (3, 5-7). 

This report presents a case of thyrolipoma in a 69-yr-old female presenting with a 5-yr history of neck enlargement and respiratory distress. Differential diagnosis of the condition is also discussed.

## Case report

A 69-yr-old female presented with a 5-yr history of neck swelling caused by thyroid enlargement that had rapidly increased in size over the previous several months. She complained of orthopnea and dyspnea on exertion and was admitted for recent exacerbation of respiratory distress. The patient was a known case of hyperthyroidism on suppression therapy. No risk factor or suspicious history for amyloidosis was detected.

On physical examination, the patient had bilateral nodular enlargement of the thyroid gland. No lymphadenopathy was detected. Ultrasonography revealed that the thyroid was diffusely enlarged with a heterogeneous echotexture. Multiple echogenic lesions were found throughout the right lobe with the largest lesion measuring 1.8 × 1 cm. Thyroid scan revealed asymmetrical enlargement of the thyroid gland with a prominent cold nodule located in the lower portion of the right thyroid lobe. No retrosternal extension of the thyroid was found. Fine-needle aspiration of the right lobe showed normal follicular cells and colloid. Based on the clinical and imaging findings, a diagnosis of multinodular goiter with obstructive symptoms was made. The patient underwent a total thyroidectomy and the specimen was submitted for histopathological study. 

**Fig. 1:**
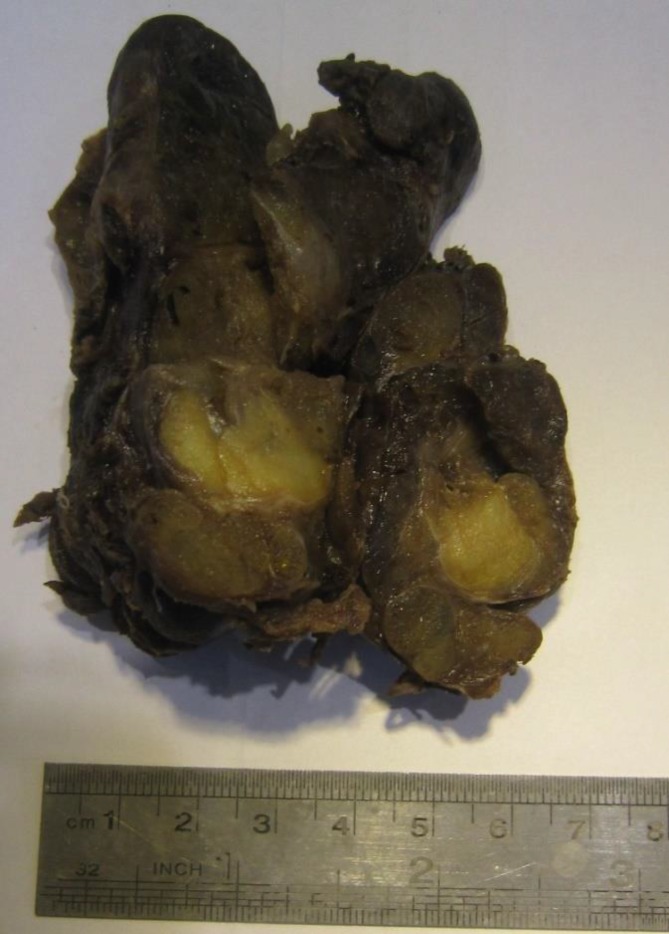
Gross resected specimen shows an enlarged thyroid gland with a yellowish nodule on the cut surfaces

**Fig 2:**
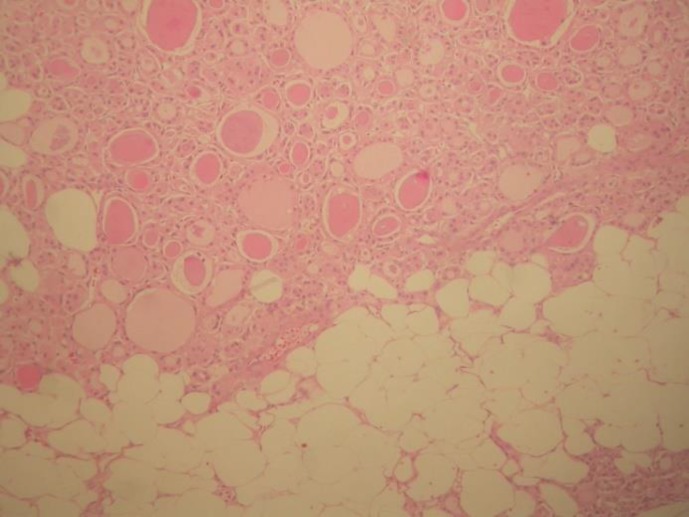
Microscopic examination of the encapsulated nodule shows the interface between thyroid follicles and mature adipose tissue (H&E staining 40x

The thyroid specimen measured 11 × 10 × 9 cm and weighed 290 gr. Cut sections showed multiple variable-sized nodules with areas of hemorrhage and cystic changes. One nodule was yellow and rubbery with a smooth regular outline measuring 2 cm at the greatest diameter in the right lower pole of thyroid (Fig. 1). Microscopic examination of the latter nodule revealed colloid-filled thyroid follicles lined by cuboidal cells with minimal variation in size in the presence of intermixed mature adipose tissue in the interfollicular stroma (Fig. 2).

The surrounding thyroid tissue showed multinodular goiter. No vascular or capsular invasion was identified. No lymphocytic infiltration, follicular destruction, or amyloid deposition was noted. No focus of papillary carcinoma was detected. A diagnosis of adenolipoma of the thyroid gland (thyrolipoma or thyroid hamartoma) in association with multinodular goiter was rendered.

## Discussion

A few adipocytes may be seen occasionally in a normal thyroid gland in subcapsular and perivascular areas or in fibrous septa; however, the presence of adipose tissue intermixed with thyroid follicles is a rare finding associated with the fat-containing lesions of the thyroid (3-5, 8). Several theories have been proposed to explain the origin of adipose tissue in the thyroid gland. Some consider these lesions to be developmental anomalies resulting from entrapment of fat tissue during encapsulation of the thyroid (1, 2, 5, 9). This theory can explain the presence of intrathyroid fat in some congenital goiters, but not in acquired lesions (5, 10). Another theory considers intrathyroid fat as stromal fibroblast metaplasia resulting from tissue hypoxia or senile involution, as in other organs (2, 4, 5, 10). The metaplasia theory explains the fat-containing lesions in certain diseases of thyroid, such as amyloidosis and colloid goiter (5). 

Differential diagnosis of fat-containing thyroid lesions includes a variety of disorders categorized as: (I) lesions containing macroscopic fat tissue, such as thyrolipoma, thyrolipomatosis, parathyroid lipoma, amyloid goiter, lymphocytic thyroiditis, Graves’ disease, encapsulated papillary carcinoma, and liposarcoma; and (II) lesions containing microscopic intracellular fat vacuoles (clear cell or lipid-rich neoplasms) (5, 9). Thyrolipoma is a well-circumscribed encapsulated follicular adenoma of the thyroid gland containing adipocytes; whereas, lipomatosis of thyroid is marked by diffuse infiltration of adipose tissue in the thyroid stroma rather than a single focus with no evidence of encapsulation (4, 9, 11). It usually presents at an earlier age as a diffusely enlarged thyroid (5, 9).

Fat-containing thyroid lesions such as thyrolipoma and thyrolipomatosis can be confused with parathyroid tissue. Parathyroid tissue could be found in the thyroid gland as a derivative of branchial pouches (4). Additionally, fat-containing thyroid lesions could be found as extrathyroidal nodules and could be mistaken for parathyroid lesions (4). While, positive immunostaining for parathormone (PTH) and the presence of cytoplasmic glycogen are seen in parathyroid tissue, positive thyroglobulin supports thyroid tissue (2, 4, 5, 12). Intrathyroid thymic lipoma is another lesion mistaken for thyrolipoma because it can present as a single thyroid nodule containing fat. In the present case, the nodule was composed of native thyroid tissue intermixed with adipocytes; thus, no origin other than thyroid was suggested. However, the diagnoses of parathyroid and intrathyroid thymic lipoma can be ruled out by a positive result of thyroglobulin immunostaining in difficult cases (5, 8). 

Heterotopic nests of adipocytes are distinguished by their limited subcapsular location (4, 12). Amyloid goiter is almost always associated with systemic amyloidosis and can be easily distinguished from other lesions by Congo red or crystal violet staining showing evident amyloid deposition (4, 12). Lipid-rich clear cell adenoma is characterized by massive steatosis of follicular cells with small round nuclei and foamy cytoplasm (4, 12). Immunostaining for thyroglobulin is useful for defining the origin of these vacuolated follicular cells (12). 

Fat infiltration can also occur in lymphocytic thyroiditis (5). Lymphocytic thyroiditis shows extensive lymphocytic infiltrate with germinal centers that destroys thyroid follicles (13). Antithyroid autoantibodies are positive in this diffuse inflammatory condition (5, 13). Malignant neoplasms can also present as thyroidal fat-containing lesions. Thyroid liposarcomas are rare and manifest as fast-growing masses with aggressive clinical features (5, 6). Encapsulated papillary carcinoma with an unusual lipomatous component in the stroma is an extremely rare tumor, only described in a few cases in the literature (5, 8). 

In the current case, microscopic examination of the resected specimen revealed multinodular goiter with a prominent well-circumscribed nodule containing follicles, lined by cuboidal cells, with minimal variation in size intermixed with mature adipocytes. There was no evidence of cytoplasmic glycogen, amyloid deposition, follicular destruction, or lymphocytic infiltration. No foci of papillary carcinoma were detected; therefore, a diagnosis of adenolipoma of the thyroid gland (thyrolipoma) in association with multinodular goiter was rendered. 

Thyrolipoma is a peculiar, rare, and benign lesion of the thyroid gland. Thyrolipoma must be considered in the differential diagnosis of any mass of the thyroid gland with an adipose component in its histology. 

## Conflict of Interests

The authors declare that there is no Conflict of Interests. 
